# Atomic Resolution Structures of Human Bufaviruses Determined by Cryo-Electron Microscopy

**DOI:** 10.3390/v10010022

**Published:** 2018-01-04

**Authors:** Maria Ilyas, Mario Mietzsch, Shweta Kailasan, Elina Väisänen, Mengxiao Luo, Paul Chipman, J. Kennon Smith, Justin Kurian, Duncan Sousa, Robert McKenna, Maria Söderlund-Venermo, Mavis Agbandje-McKenna

**Affiliations:** 1 Department of Biochemistry and Molecular Biology, University of Florida, Gainesville, FL 32611, USA; mariailyas@ufl.edu (M.I.); mario.mietzsch@ufl.edu (M.M.); shwetakailasan@gmail.com (S.K.); mxluo@ufl.edu (M.L.); pchipman@ufl.edu (P.C.); jkennonsmith@ufl.edu (J.K.S.); justinkurian@ufl.edu (J.K.); rmckenna@ufl.edu (R.M.); 2 Center for Structural Biology, The McKnight Brain Institute, University of Florida, Gainesville, FL 32611, USA; 3 Department of Virology, University of Helsinki, P.O. Box 21 (Haartmaninkatu 3), University of Helsinki, FIN-00014 Helsinki, Finland; elina.vaisanen@helsinki.fi (E.V.); Maria.Soderlund-Vernemo@Helsinki.fi (M.S.-V.); 4 Biological Science Imaging Resource, Department of Biological Sciences, The Florida State University, 89 Chieftan Way, Rm 119, Tallahassee, FL 32306, USA; dsousa@fsu.edu

**Keywords:** parvoviruses, bufavirus, single-stranded DNA virus, cryo-EM and image reconstruction

## Abstract

Bufavirus strain 1 (BuV1), a member of the *Protoparvovirus* genus of the *Parvoviridae*, was first isolated from fecal samples of children with acute diarrhea in Burkina Faso. Since this initial discovery, BuVs have been isolated in several countries, including Finland, the Netherlands, and Bhutan, in pediatric patients exhibiting similar symptoms. Towards their characterization, the structures of virus-like particles of BuV1, BuV2, and BuV3, the current known genotypes, have been determined by cryo-electron microscopy and image reconstruction to 2.84, 3.79, and 3.25 Å, respectively. The BuVs, 65–73% identical in amino acid sequence, conserve the major viral protein, VP2, structure and general capsid surface features of parvoviruses. These include a core β-barrel (βB-βI), α-helix A, and large surface loops inserted between these elements in VP2. The capsid contains depressions at the icosahedral 2-fold and around the 5-fold axes, and has three separated protrusions surrounding the 3-fold axes. Structure comparison among the BuVs and to available parvovirus structures revealed capsid surface variations and capsid 3-fold protrusions that depart from the single pinwheel arrangement of the animal protoparvoviruses. These structures provide a platform to begin the molecular characterization of these potentially pathogenic viruses.

## 1. Introduction

Advances in DNA sequencing technology have led to the discovery of several new members of the ssDNA *Parvoviridae* that infect humans, including human bocaviruses 1 to 4 (HBoV1-HBoV4), cutavirus (CuV), tusavirus (TuV), bufavirus 1 to 3 (BuV1-BuV3), and human parvovirus 4 (PARV4) [1,2,3,4,5,6,7]. These viruses are grouped into different genera based on the DNA sequence of their non-structural (NS) proteins [8]. The BuVs, along with CuV and TuV belong to the genus *Protoparvovirus*, which prior the BuV discovery contained only animal (non-human) infecting viruses, and are classified as novel *Primate protoparvovirus* 1 species. The HBoVs are also the only human-infecting members of *Bocaparvovirus*. PARV4 is currently the only human-infecting member of the novel *Tetraparvovirus* genus [8].

To date, information on BuVs remains epidemiological. BuV1 was isolated in 2012 from fecal samples of children with acute diarrhea in Burkina Faso, West Africa [4]. Additional genotypes, BuV2 and BuV3, which are antigenically distinct from BuV1 or each other, and thus considered serotypes, have been observed in pediatric patients with gastrointestinal symptoms [9,10,11,12]. BuV2 was discovered also in Burkina Faso, while BuV3 has been observed in other countries, including Bhutan, China, Finland, Netherlands, Thailand, and Turkey [4,9,10,11]. This includes detection in nasal swabs in a low percentage (0.1%) of pediatric samples in Finland [12]. BuV has not been detected in non-diarrheal feces of healthy individuals. In addition, there are cases with no other known pathogens detected. However, the viral loads are low and the prevalence among diarrheic patients is low. These observations indicate that BuVs are putative pathogens, but it is currently unclear whether BuVs are the sole causative agents of the symptoms associated with detection or are accidental passengers with other bacterial and viral gastrointestinal pathogens [12].

The *Parvorividae* are small, non-enveloped viruses assembled with T-1 icosahedral symmetry. They are divided into two subfamilies, *Parvovirinae* and *Densovirinae* based on infection of vertebrates and invertebrates, respectively. Members of the *Protoparvovirus* genus belong to the *Parvovirinae* subfamily and package genomes of ~5 kb [8]. As observed for other protoparvoviruses, the three BuVs have two major open reading frames (ORFs) under the control of the p6 and p38 promoters and responsible for encoding the non-structural (NS, left ORF) and capsid viral proteins (VPs, right ORF), respectively [4,8,13]. The VP ORF encodes for two overlapping structural proteins, VP1 and VP2, sixty copies of which assemble their T = 1 icosahedral capsid in a predicted 1:10 ratio. The VP1, at 80.4 kDa, is the larger minor capsid component containing an N-terminal extension termed VP1u compared to VP2. The VP2 is 64.5 kDa, and is the major capsid component. The amino acid sequence of this protein is 64%, 73%, and 65% identical between BuV1 and BuV2, and BuV1 and BuV3, BuV2 and BuV3, respectively [4,9,10,11]. A small ORF located in the center of the BuV genome encodes a potential ~130 amino acid protein [4]. No equivalence has been found for this ORF in other parvoviruses. The ORF reported to express the small alternatively translated protein in other protoparvoviruses and amdoparvoviruses has not been observed in BuVs [14]. The VP1 sequence of BuV has an overall amino acid sequence identity of 10–31% to other *Parvovirinae* VP1s [4].

The parvovirus capsids are known to be multi-functional and to participate in various steps during infection. They carry determinants of host/tissue cell recognition, endosomal trafficking, capsid assembly, genome packaging, and antibody recognition [15]. The determinants of these functions within the BuV capsid are yet to be defined. To begin characterization of their capsid structure, virus-like particles (VLPs) of BuV1, BuV2, and BuV3, assembled from the major coat protein VP2, were produced and purified for structure determination by cryo-electron microscopy (cryo-EM) and image reconstruction (cryo-reconstruction). For each BuV1, BuV2, and BuV3 genotype, the structures were determined to 2.84, 3.79, and 3.25 Å resolution, respectively, and the VP2 structure ordered from residues 33 to 568, 33 to 567, and 33 to 572, respectively. This range of ~540 ordered residues is consistent with those of crystal and/or cryo-reconstructed structures available for other parvoviruses, including members of the protoparvoviruses [16,17,18,19,20,21]. The VP structure conserves the β-barrel (βB–βI) and α-helix A observed for all parvovirus structures. Large loops inserted between these secondary structure elements provide the surface morphology of the capsid, including the conserved depressions at the icosahedral 2-fold axes and surrounding a channel at the 5-fold axes. However, unlike the animal protoparvoviruses, the bufavirus capsid contain three separated protrusions surrounding the 3-fold axes, as has been reported for members of the *Dependoparvovirus* genus, human parvovirus B19, and the HBoVs [22,23,24]. Structure comparison among the BuV strains and to other available *protoparvovirus* structures revealed capsid surface variations in previously defined common variable regions (VRs) associated with serotype and strain specific functions. These include receptor attachment, cellular trafficking, genome transcription, and antigenic reactivity. These structures thus provide a 3D platform to begin functional annotation of these potential pathogens.

## 2. Materials and Methods

### 2.1. Production and Purification of BuV1, BuV2, and BuV3 Virus-Like Particles

The BuV1, BuV2, and BuV3 VP2 genes were cloned into the pFastBac1 plasmid to produce a recombinant baculovirus that expresses virus-like particles (VLPs) using the Bac-to-Bac protocol according to the manufacturer’s instructions (Invitrogen, Carlsbad, CA, USA) [12]. *Sf9* insect cells (ATCC), maintained in Grace’s medium (Invitrogen) with 10% FBS (fetal bovine serum) and antibiotics, were infected with the recombinant baculoviruses and harvested 72 h post infection by centrifugation at 3000 rpm on Beckman JA-20 (Beckman Coulter, Indianapolis, IN, USA) for 20 min at 4 °C. The pellets were re-suspended in lysis buffer (50 mM Tris-HCl, pH 8.0, 100 mM NaCl, 1 mM EDTA (ethylenediaminetetraacetic acid), 0.2% Triton X-100) (TNET buffer) and frozen at −20 °C until purification. For purification, VLPs were released from the infected cell pellet by three freeze-thaw cycles. Benzonase (Millipore, Novagen, Burlington, MA, USA) was added after the second freeze-thaw cycle and the sample was incubated for 30 min at 37 °C. One microliter of benzonase per 10 mL of pellet supernatant (activity of 1 × 10^6^ U/mg of protein) with 2 mM MgCl_2_ was used for this reaction. The samples were centrifuged twice at 10,000 rpm (Beckman JA-20) for 15 min at 4 °C to remove any large cellular debris that remained recalcitrant to resuspension. The resulting clarified supernatant were subjected to a 20% (*w*/*v*) sucrose cushion (prepared in the TNET buffer) to pellet the VLPs by ultracentrifugation at 45,000 rpm (Beckman 70-Ti) for 3 h at 4 °C. The resulting pellets were resuspended overnight in 25 mM Tris-HCl, 100 mM NaCl, 0.2% Triton X-100, and 2 mM MgCl_2_ (TNTM buffer). The samples were further purified using sucrose gradients (5 to 40% sucrose in TNTM) ultracentrifugation at 35,000 rpm (SW40-Ti, Beckman Coulter, Indianapolis, IN, USA) for 3 h at 4 °C. Visible blue VLP bands were extracted at 20% for all three viruses. Equilibrium dialysis was performed against 1× phosphate buffer saline (PBS) (2.8 mM KCl, 137 mM NaCl, 10 mM Na_2_HPO_4_, 1.8 mM KH_2_PO_4_) at 4 °C. Concentrations (in mg/mL) were determined based on UV absorbance of 280 nm with an extinction coefficient of 1.7 M^−1^·cm^−1^. The purified virus samples were concentrated to ~0.5 mg/mL using Amicon concentrators (EMD Millipore, Darmstadt, Germany) for further characterization and structure determination.

### 2.2. VLP Sample Purity and Integrity

The purity and integrity of the VLPs were confirmed by sodium dodecyl sulfate polyacrylamide gel electrophoresis (SDS-PAGE) and negative-stain electron microscopy (EM), respectively. For the SDS-PAGE analysis, the samples were incubated with 1× Laemmle Sample Buffer (Bio-Rad, Hercules, CA, USA) with 10% β-mercaptoethanol and boiled for 10 min at 100 °C. The denatured proteins were applied to a 12% polyacrylamide gel and ran at 120 V. The gel was washed three times with distilled water (diH_2_O) and stained with GelCode Blue Protein Safe stain (Invitrogen, Carlsbad, CA, USA) for 30 min. The gel was de-stained with diH_2_O prior to imaging using a GelDoc EX system (Bio-Rad). For negative stain EM, carbon-coated copper EM grids (Ted Pella, Redding, CA, USA) were incubated with 5 µL of sample for 1–5 min, washed with diH_2_O, and stained with Nano-W (Nanoprobes, Inc., Yaphank, NY, USA) for 30 s. The grids were imaged on a Tecnai G2 Spirit TEM (FEI Co., Hillsboro, OR, USA) microscope operated at an accelerating voltage of 120 kV and micrographs were collected on a Gatan 2 K × 2 K CCD camera (Gatan, Inc., Pleasanton, CA, USA).

### 2.3. Stability of BuV VLPs

The thermal stability of purified and intact BuV VLPs was determined by differential scanning fluorimetry (DSF) as previously described [25]. Briefly, BuV VLPs, extracted from the 20% sucrose fraction, at a concentration of 0.2 mg/mL, were dialyzed or buffer exchanged into citrate-phosphate buffer at pH 7.4. To each sample, 2.5 μL of 1% SYPRO-Orange dye (Molecular Probes, Invitrogen, Carlsbad, CA, USA) was added for a final reaction volume of 25 μL. Adeno-associated virus serotype 5 (AAV5) VLPs with a published T_m_ of 89.2 ± 0.0, was used as a positive control [25]. The heating assay was conducted in a thermocycler (BioRad CFX Connect) with the temperature ramped from 30 to 99 °C at 0.1 °C/6 s. The melting temperature (T_m_) for each virus was defined as the vertex of the first derivative (dF/dT) of relative fluorescence unit (RFU) values.

### 2.4. Cryo-Electron Microscopy Data Collection and Image Reconstruction

Three microliters each of the BuV VLP samples were applied to glow-discharged Quantifoil copper grids with 2 nm continuous carbon support over holes (Quantifoil R 2/4 400 mesh) or C-flat holey carbon grids (Electron Microscopy Sciences, 2/2 400 mesh, Hatfield, PA, USA), blotted, and vitrified using a Vitrobot Mark 4 (FEI) at 95% humidity and 4 °C. The particle distribution and ice quality of the grids were screened in-house using an FEI Tecnai G2 F20-TWIN microscope (FEI Co., Hillsboro, OR, USA) operated under low-dose conditions (200 kV, ~20 e^−^/Å^2^). Images were collected on a Gatan UltraScan 4000 CCD camera (Gatan, Inc., Pleasanton, CA, USA). Grids deemed optimal for data collection were used for collecting micrograph movie frames using the Leginon semi-automated application [26] on a Titan Krios electron microscope (FEI Co.) operated at 300 kV with images recorded on a Gatan K2 Summit direct electron detection camera for BuV1 and BuV3. The microscope was equipped with a Gatan post-column imaging filter (GIF) utilizing a slit width of 20 eV. Data collection used counting mode and an accumulated dose of 75 e^−^/Å^2^ fractionated into 50 or 60 movie frames per micrograph. Movie frames alignment used the MotionCor2 application with dose weighting [27]. These data sets were collected as part of the National Institutes of Health (NIH) “West/Midwest Consortium for High-Resolution Cryo Electron Microscopy” project. A nominal magnification of 130,000× was used for data collection resulting in a pixel size of 1.1 Å. The BuV2 data set was collected on a Titan Krios electron microscope (FEI Co.) operated at 300 kV using a DE20 (Direct Electron, San Diego, CA, USA) direct electron detector operated under super-resolution mode. A dose of 57 e^−^/Å^2^ was fractionated over 34 movie frames per micrograph. Movie frame alignment used the DE_process_frames software package (Direct Electron) without dose weighting as previously described [24,28]. The micrographs were collected at a magnification of 29,000 resulting in a pixel size of 1.22 Å. The BuV2 data set was collected as part of the NIH “Southeastern Center for Microscopy of MacroMolecular Machines (SECM4)” project. The data collection parameters for all three data sets are provided in Table 1.

Individual particle images were selected from the aligned micrographs using the automated particle picking option (M) within the AUTOPP subroutine in the AUTO3DEM program [29] for BuV1 or semi-automated boxing using the e2boxer subroutine in the EMAN2 program [30] for BuV2 and BuV3. The AUTOPP subroutine (options F and O) of AUTO3DEM was used for pre-processing (normalization and apodization) of the extracted particle images [29]. The three structures were determined utilizing the gold standard protocol in AUTO3DEM [29]. The defocus values for each micrograph were estimated using the CTFFIND4 program to correct microscope-related contrast transfer functions (CTFs) [31]. Initial models (~30 Å resolution) used to search the particle origins and orientations for each data set were generated from 100 particle images using the ab initio model generating subroutine within AUTO3DEM while applying icosahedral symmetry. This was followed by cycles of origin and orientation refinement, solvent flattening, and CTF refinement. To minimize the effects of drift in early movie frames and potential radiation damage in latter movie frames, the movie frames were re-aligned while truncating the number of frames, utilizing frames 3 to 20 (BuV1 and BuV3) or frames 4 to 30 (BuV2). The particle origin and orientation information from the CTF refinement cycle were applied to the truncated frame particle images and used for further rounds of origin and orientation refinement, followed by removal of outlier particles based on deviation from refined origins and orientations with the “score fractions” option within AUTO3DEM. Different B-factor (temperature factor) values, 1/50, 1/100, 1/150, and 1/200 Å^2^, were applied to sharpen the high resolution features of the resulting final maps followed by visual inspection in the Coot and Chimera programs [32,33]. All four maps were used during model building, with the 1/200 Å^2^ B-factor corrected map highlighting amino acid side chains for highly flexible surface loops, while the 1/50 Å^2^ B-factor corrected map highlighted ordered main chain. The resolution of the cryo-reconstructed maps for BuV1, BuV2, and BuV3 were estimated to be 2.84, 3.79, and 3.25 Å, respectively, based on a Fourier Shell Correlation (FSC) of 0.143 (Table 1).

### 2.5. Model Building and Structure Refinement

The cryo-reconstructed density maps were interpreted using three-dimensional (3D) models of the VP2 of BuV1, BuV2, and BuV3 generated with the SWISS Model online server (https://www.swissmodel.expasy.org/) from their respective VP2 sequence (NCBI accession# AIU47674, AFN44277, and BAO56913) and the atomic coordinates of minute virus of mice strain p (MVMp) VP2 (RCSB PDB ID: 1Z14) supplied as a template [20,34]. These VP2 models were used to generate all-atom 60-mer capsid models using the online VIPERdb Oligomer Generator by icosahedral matrix multiplication for a T = 1 capsid (http://viperdb.scripps.edu) [35]. In preparation for model fitting, the cryo-reconstructed density maps were converted from the Purdue Image Format (PIF) to an XPlor format using the “e2proc3D.py” subroutine in EMAN2 [30]. The 60-mer capsid models were then docked into the maps using the ‘Fit-in-map’ function in the Chimera program by rigid body rotations and translations [33]. During the model fitting, the voxel (pixel) size of each map was surveyed to optimize the correlation coefficient (CC) between the models and maps. The fitted models were saved related to the respective map. Each map was then converted to a CCP4 format using the program MAPMAN and resized to the voxel size determined in Chimera [33,36] (Table 1). The reference VP2 monomer was extracted from each 60-mer and the side- and main-chains adjusted into the maps by manual building and the real-space-refinement subroutine in the Coot program [32]. VP2 residues 33 to 568, 33 to 567, and 33 to 572 were ordered for BuV1, BuV2, and BuV3, respectively, at a sigma (σ) threshold of ≥1.0.

The Non-Crystallographic Symmetry (NCS) extension in the Coot program was used to generate a 60-mer capsid from the adjusted VP2 models for further refinement. The capsid was refined against the 1/50 Å^2^ B-factor corrected map utilizing the rigid body, real space, and B-factor refinement subroutines in the Phenix program [37]. Model refinement was alternated with visualization within the maps and adjustment in Coot to maintain model geometry as well as rotamer and Ramachandran constraints [31]. The CC and refinement statistics, including root mean square deviations (RMSD) from ideal bond lengths and angles (Table 1), were analyzed by Phenix [37]. The secondary structure elements were assigned based on the phi and psi angles of the VP2 monomer extracted from the refined capsid 60-mer.

### 2.6. Structure Comparison among the BuV Serotypes

The reconstructed density maps and the respective BuV VP2 models built into them were compared to identify regions of structural similarities and differences. The surface morphology of the maps were visually compared using Chimera while the VP2 models were superposed in Coot to obtain overall and paired RMSDs between Cα positions. Deviations between non-overlapping Cα positions, due to residue deletion/insertions, were measured using the distance tool in Coot [32]. Regions of two or more adjacent amino acids with ≥2.0 Å difference in superposed VP2 Cα position were considered to be structurally diverse and assigned to previously described VRs [18,38].

### 2.7. Comparison of BuV1 to Other Parvovirus Capsid Structures at the Genus and Subfamily Levels

The VP2 sequences of *Protoparvovirus* members for which structures are available, canine parvovirus (CPV), feline panleukopenia virus (FPV), H-1 parvovirus (H-1PV), LuIII, MVMp, and porcine parvovirus (PPV), were compared to BuV1 by pairwise alignment using the online Pairwise Sequence Alignment tool at the EMBL-EBI (https://www.ebi.ac.uk/Tools/psa/) [39]. Default settings were used. For structural comparison, the VP2 structures of these viruses (PDB IDs: CPV, 2CAS; FPV, 1C8E; H-1PV, 4GBT; LuIII, 6B8Q; MVM, 1Z14; PPV, 1K3V) [16,17,18,19,20,21] were compared to the BuV1 VP2 structure by secondary structure matching (SSM) in the PDBeFOLD program [39]. Distance differences at regions of non-overlapping Cα positions, due to deletions and insertions, were measured as described above. Variable regions among the protoparvoviruses were assigned as previously described [18,38]. At the subfamily level, the VP2 sequences and structures available for *Parvovirinae* members that infect humans, from three different genera, e.g., Adeno-associated virus serotype 2 (AAV2) (*Dependoparvovirus*), human parvovirus B19 (B19) (*Erythroparvovirus*), and human bocaparvovirus 1 (HBoV1) (*Bocaparvovirus*), were compared to BuV1 (*Protoparvovirus*). Similar to the *Protoparvovirus* comparison above, pairwise sequence alignment and SSM were used. The Chimera program was used to visualize the capsid surface morphology similarities and differences for the genus and subfamily level viruses compared. For this analysis, the capsid 60-mers were generated using the VIPERdb online server (http://viperdb.scripps.edu) as described above [35].

### 2.8. Structure Accession Numbers

The BuV1, BuV2, and BuV3 cryo-EM reconstructed density maps and models built for their capsids were deposited with accession numbers EMD-7300 and 6BWX, EMD-7301 and 6BX0, and EMD-7302 and 6BX1, respectively, in the Electron Microscopy Data Bank (EMDB).

## 3. Results and Discussion

### 3.1. Purified BuV VLPs Are Suitable for Atomic Resolution Structure Determination

**Figure 1:**
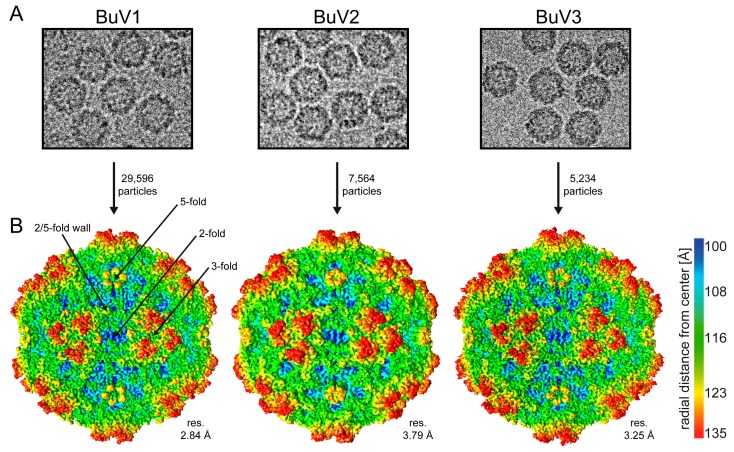
The capsid structure of the BuVs. (**A**) Example virus particles from cryo-electron micrographs; (**B**) cryo-reconstructed capsid density map of BuV1, BuV2, and BuV3 contoured at a sigma (σ) threshold of 1.0. The icosahedral 2-, 3-, and 5-fold axes and the 2/5-fold wall are indicated on the BuV1 map. The reconstructed maps are colored according to radial distance from the particle center (blue to red), as indicated by the scale bar (right). This figure was generated using Chimera [33].

**Figure 2:**
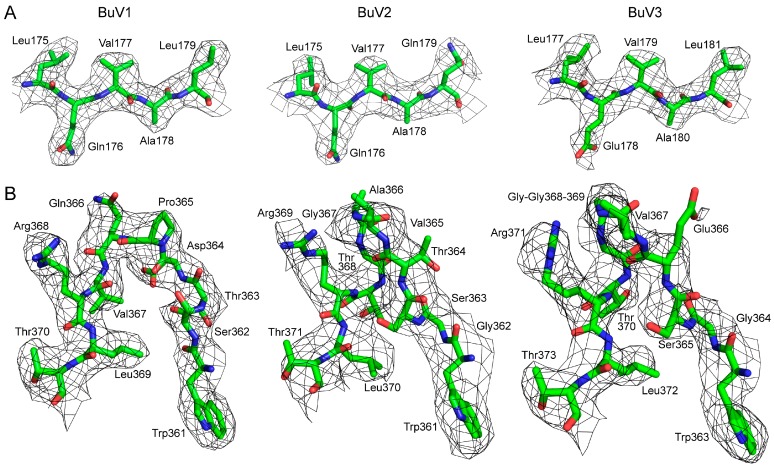
The BuV density maps and atomic models. (**A**) Example densities for BuV1, BuV2, and BuV3 in the core βE strand; (**B**) the density of variable region (VR)-VI for BuV1, BuV2, and BuV3 with the modeled residues. At the apex of the loop, BuV1 has a one residue deletion compared to BuV2 and BuV3. The amino acid residues are shown by stick representation and colored according to atom type: C = green, O = red, N = blue inside a black mesh density map. Residues are as labeled. This image was generated using PyMOL (DeLano, W. L. (2002). PyMOL. DeLano Scientific, San Carlos, CA, USA, 700).

**Figure 3:**
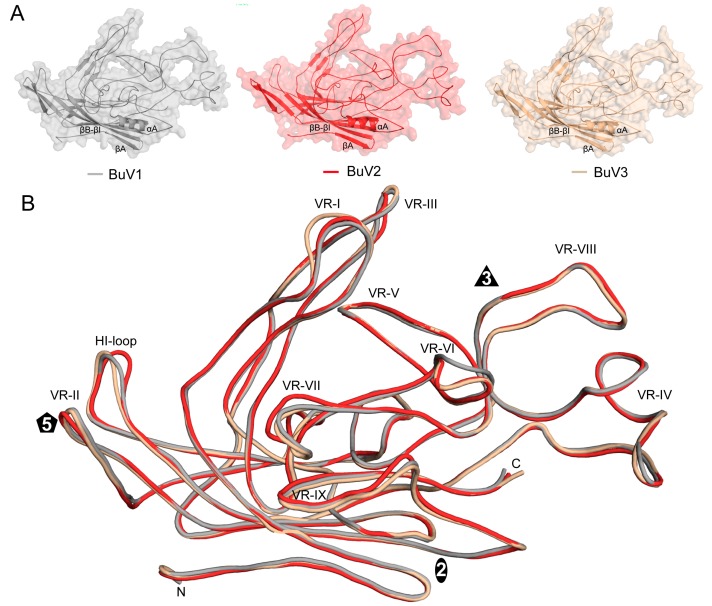
The BuV VP2 structure. (**A**) Cartoon diagrams of the BuV VP2 structures within a transparent surface representation. The conserved β-barrel core motif (βB–βI), βA, and the αA helix are indicated; (**B**) structural superposition of BuV1 (gray), BuV2 (red), and BuV3 (tan) VP2 shown as coil diagrams with the position of VR-I to VR-IX, labeled. The approximate icosahedral 2-, 3-, and 5-fold axes are represented by an oval, triangle, and pentagon, respectively. This image was generated using PyMOL (DeLano, W. L. (2002). PyMOL. DeLano Scientific, San Carlos, CA, USA, 700).

**Figure 4:**
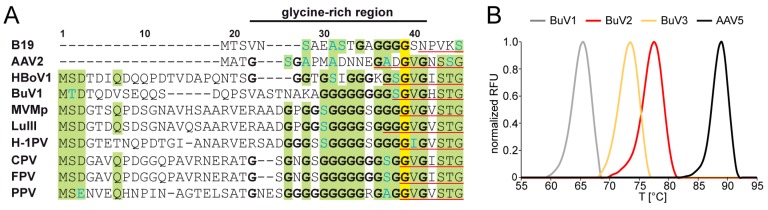
(**A**) Sequence alignment of the VP2 or VP3 N-terminus of selected members of the *Parvovirinae* subfamily for which 3D VP structures are available. Amino acids highlighted in yellow are 100% identical, in green ≥60% identical. Residues colored in blue indicate similar amino acids in conserved regions. Glycines within the glycine-rich region are shown in bold. The N-terminus of the reported VP structures are indicated by the underlined (red) sequence. Amino acids further N-terminal are not ordered in these virus structures; (**B**) thermal profile (shown as normalized relative fluorescence unit (RFU)) versus temperature (°C) of BuV1, BuV2, BuV3, and AAV5 obtained by differential scanning fluorimetry (DSF) analysis. A representative profile is shown for each serotype. Each profile is colored according to the serotype, as shown above the plot.

The VP2 of BuV1, BuV2, and BuV3, produced using the baculovirus/*Sf*9 protein expression system and purified using sucrose cushions and gradients, assembled VLPs suitable for structure determination by cryo-reconstruction (Figure 1A). Movie frame micrographs collected for these VLPs, at two different cryo-EM resources (West/Midwest Consortium for High-Resolution Cryo Electron Microscopy and SECM4) yielded capsid structures to 2.84, 3.79, and 3.25 Å resolution from 29,596, 7564, and 5234 particle images, respectively, for BuV1, BuV2, and BuV3 (Table 1, Figure 1B). Differences in resolution of the BuV structures are likely due to micrograph quality and the different Å/pixel used for the different data collections, for example, 1.07 Å for BuV1, 1.06 Å for BuV3, and 1.22 Å for BuV2 (Table 1). The capsid density morphology exhibited the known surface features of other *Parvovirinae* subfamily members. These included shallow depressions at the icosahedral 2-fold axes and surrounding the 5-fold axes, and three protrusions surrounding each 3-fold axis (Figure 1B). A raised capsid region between the depressions at the 2- and 5-fold axes is referred to as the 2/5-fold wall (Figure 1B). Qualitative visual inspection of the capsid surfaces indicated differences on raised regions, likely due to amino acid side-chain sequence and structure variation. At these resolutions, the VP2 sequence of each BuV genotype was readily interpretable within the cryo-reconstructed density maps for core and surface loop regions (Figure 2). For each BuV, the first 32 N-terminal residues of VP2 were disordered, and thus residues modeled for BuV1, BuV2, and BuV3 were 33–568, 33–567, and 33–572, respectively, with the last residue being their C-terminus (Figure 3). This N-terminal disorder is consistent with observations for all structures previously determined for members of the *Parvoviridae* with the exception of B19 for which the N-terminus of VP2 was exposed on the capsid surface of a medium resolution cryo-reconstructed density map [15,40]. The N-terminal disorder has been proposed to be due to flexibility of this VP region arising from a long stretch of glycine residues in the parvoviruses (Figure 4A) [24]. Thus, density ordering, regardless of the method utilized for structure determination (X-ray crystallography or cryo-reconstruction), generally begins towards the end of this glycine stretch (Figure 4A).

The model building for the ordered density was conducted at a σ ≥ 2, however, two surface loops, residues 381 to 392, 381 to 392, 384 to 395 and 496 to 503, 495 to 502, and 499 to 506 in BuV1, BuV2, and BuV3, respectively, were less ordered and were built at 1σ. For BuV2, at the lowest resolution of 3.79 Å, only the main chain for the residues 381–391 could be interpreted with confidence. Notably, as has been reported for other cryo-reconstructed density maps, a number of the acidic residue side-chains were less ordered because of their increased sensitivity to electron induced radiation damage (e.g., Glu366 in Figure 2B). The final capsid models were refined with a good correlation coefficient (relative to the cryo-reconstructed maps) and geometries consistent with other virus structures, including parvoviruses, determined to a similar resolution (Table 1) [35].

The VP2 monomer extracted from the refined 60-mer capsids of the BuVs contained the core β-strands (βA–βI) and α-helix A (αA) observed in other parvoviruses and large loop insertions between these secondary structure elements (Figure 3). Superposition of the VP2 structures from the three BuVs showed the structural conservation of the core and most of the loop structures. Conformational divergence was only observed at the apexes of some of the surfaces loops in previously defined VRs.

As previously mentioned, the VP2 sequence of the BuVs shares 64%, 73%, and 65% sequence identity between BuV1 and BuV2, BuV1 and BuV3, and BuV2 and BuV3, respectively. This level of identity results in a VP2 topology that is highly superposable with 498/536 (~92.9%), 528/536 (98.5%), and 508/535 (95.0%), respectively, of their pairwise Cα positions aligned consistent with an anticipated high structure similarity. The structures differ from each other at seven surface loop regions located within previously defined VRs (Figure 3B). These loops are located at the 2-fold wall (VR-VI), 2/5-fold wall (VR-IV, VR-VII, and VR-IX), and the protrusions surrounding the 3-fold axes (VR-I and VR-III). Minor changes were observed in the 5-fold region at the tops of the DE loops forming the 5-fold channel (VR-II) and the HI loop that lines the floor of the channel surrounding this channel. VR-IX is mostly structurally conserved among all three viruses, with only the BuV2 residue 540 being conformationally different, VR-VI and VR-VII adopt different conformations, with BuV1 differing from BuV2 and BuV3 at VR-VI and BuV2 differing from BuV1 and BuV3 at VR-VII. In VR-I, two additional residues are inserted in BuV3 relative to BuV1 and BuV2 and VR-III contains conformational difference among all three BuV resulting in Cα position differences of up to 5.0 Å (Figure 3B). These two VRs extend from the 5-fold region to assemble the 3-fold protrusions along with VR-IV and VR-VIII that are structurally conserved. These four loops are contributed from two VP2 monomers with one contributing VR-I and VR-III and the other VR-IV and VR-VIII. At the top of the 5-fold DE loop, BuV2 residue 160 adopts a different conformation to BuV1/BuV3 residues 161/163 that are structurally similar. This structural difference of BuV2 likely stems from four amino acid changes in the DE loop compared to BuV1 and BuV3 that share the same amino acid composition. The HI loop adopts a different conformation in all three viruses. Although notably, as mentioned above, the HI loop residues are less ordered than other VP2 regions and side chain density was difficult to interpret for residues 498–506. The main chains were interpreted with confidence. All the loop differences give rise to localized capsid surface morphology differences between the BuVs at the 2/5-fold wall and the regions surrounding the 3-fold and 5-fold axes (Figure 1B). BuV2, which is the most different, has more rounded 3-fold protrusions due to VR-I and VR-III and 2/5-fold wall is thicker and more pronounced than BuV1 and BuV3 due to the larger VR-VII (Figure 1B). Significantly, the 2/5-fold wall and 3-fold protrusions are reported antigenic hotspots for the parvoviruses [41,42,43,44]. These structural differences thus support the reported lack of serum cross-reactivity for these viruses and the suggestion that they are serotypes [12].

### 3.2. A Unique Disulfide Bond in BuV2 May Confer Increased Capsid Stability

The melting temperature (T_m_s) of the BuV capsids determined by DSF were 64.9 ± 0.2 °C for BuV1, 77.3 ± 0.3 °C for BuV2, and 73.4 ± 0.2 °C for BuV3 (Figure 4B). The T_m_ for AAV5 was consistent with the published data at 88.9 ± 0.2. The T_m_ values obtained for BuV2 and BuV3 were within the range reported for other members of the *protoparvovirus* genus, for example, MVM [45]. Interestingly, the ratio of basic and acidic amino acids in the BuV1, BuV2, and BuV3 capsids of 0.78 (45/58), 0.7 (45/58), and 0.72 (39/54) correlated with the determined T_m_ value. This trend of a higher capsid stability for viruses with a lower basic/acidic residue ratio has also been observed when 10 different AAV serotypes were analyzed [25]. The exact reason for this observation is not known.

**Figure 5:**
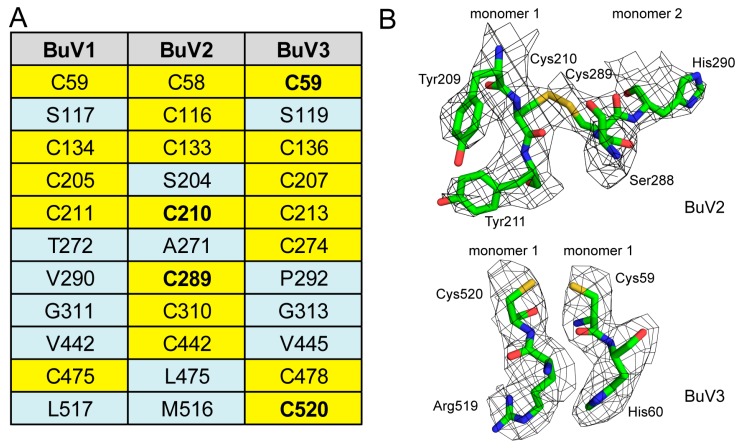
Cysteines in BuV capsids. (**A**) Table of all cysteines (highlighted in yellow) in BuV1, BuV2 and BuV3 VP2; (**B**) density maps and atomic models for the potential disulfide bonds (highlighted in bold in A) in BuV2 (top) and proximal cysteine residues in BuV3 (bottom). The amino acid residues are shown as stick representation and colored according to atom type: C = green, O = red, N = blue inside a black mesh density map. This image was generated using PyMOL (DeLano, W. L. (2002). PyMOL. DeLano Scientific, San Carlos, CA, USA, 700).

Towards identifying other potential contributors within the VP2 sequence and structure responsible for this disparity in stability between BuV1 and the other two viruses, the number and interactions of cysteine residues within their sequence were analyzed. BuV1 contains five cysteine residues while BuV2 and BuV3 contain seven each (Figure 5A). Interestingly, BuV2, has a disulfide bond between residues C210 and C289 of 3-fold related VP2 monomers (Figure 5B). The residues are located below the section of VR-IV positioned on the 2/5-fold wall. This interaction is not possible for BuV1 and BuV3 which conserve the C210 equivalent residue position but have V290 and P292, respectively, at positions equivalent to BuV2 C289. The BuV2 disulfide bond interaction could contribute to the increased capsid stability observed. BuV3 does contain two cysteine residues, C59 and C520 within the same VP2 monomer, at a distance of 3.45 Å, positioned to interact within a hydrophobic pocket, but they are not engaged in a disulfide bond (Figure 5B). This proximity may be contributing to the stable BuV3 phenotype compared to BuV1. Significantly, in all the structures determined to date for parvoviruses, either by X-ray crystallography or cryo-reconstruction, there has been no disulfide bond observed, despite the fact that cysteine residues have been positioned within interacting distance, similar to BuV3 C59 and C520 as mentioned above. In some examples, the side-chain of the residues are rotated away from each other. Thus, a role for this unique BuV2 disulfide bond beyond the potential contribution to increased capsid stability remains to be determined.

### 3.3. BuV1 VP2 and Capsids Conserve Protoparvovirus Features Despite Low Sequence Identity But Have a Unique 3-Fold Morphology

**Figure 6:**
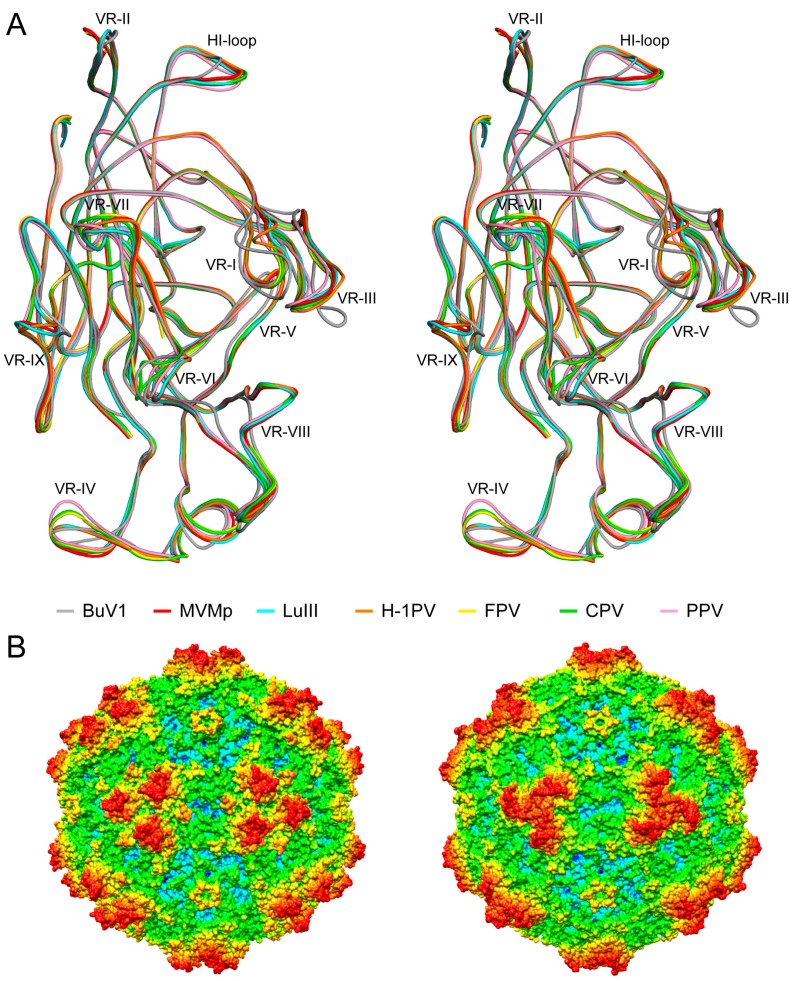
The structures of the protoparvoviruses. (**A**) Stereo projection of a structural superposition of available protoparvovirus structures onto BuV1 shown in coil representation. Individual colors are as indicated. The VR regions, VR-I to VR-IX, and the HI-loop are shown. Figure generated using PyMOL (DeLano, W. L. (2002). PyMOL. DeLano Scientific, San Carlos, CA, 700); (**B**) radially-colored capsid surface representations (blue to red, as shown in Figure 1) of the BuV1 (left) and MVMp (right) capsid models viewed along the 2-fold axis. This image was generated using Chimera [33].

The BuV1 VP2 sequence and structure as well as capsid structure was compared to other protoparvoviruses with available 3D structures. The sequence identity among these protoparvoviruses ranged from ~29% between BuV1 and MVMp and ~99% between CPV and FPV with the rest at ~50 to ~70% (Table 2). Despite this disparate range of identity, the viruses shared high structural similarity, ranging from ~74% between BuV1 and H-1PV up to 98% between other viruses (Table 2). The Cα positions of the core β-strands (βA–βI) and αA residues, accounting for ~20% of the VP2 sequence, were completely superposable for the protoparvoviruses, while the loops between these secondary structure elements follow the same topology, including for BuV1 (Figure 6). The lower sequence identity of BuV1 to the other protoparvoviruses resulted in it possessing the most divergent surface loop structures compared to the other viruses (Figure 6A). The largest of these conformational differences was at VR-VIII, although noticeable differences also occurred at other VRs, for example, VR-I, VR-III, VR-VI, and VR-VII (Figure 6A). The BuV1 VR-VIII loop difference to the other protoparvovirues stemmed from a deletion of ~11 residues compared to the other viruses (Figure 6A). This deletion removed the residues closest to the three-fold axes, resulting in three separate protrusions (also in BuV2 and BuV3) compared to the other protoparvoviruses that have a single pinwheel protrusion centered at this axis (Figure 6B). Additional differences on the 3-fold protrusions included residue insertions and conformational differences in VR-I and VR-III. At the 2-fold region, the depression was conserved while VR-IX adopted a different conformation; on the 2-fold wall, VR-VI was larger in BuV1 due to an 8-residue insertion. At the 2/5-fold wall, VR-VII of BuV1 had a two-residue deletion and was conformationally different (Figure 6A). Significantly, the two-fold region of MVM and CPV was reported to serve as the recognition site for sialic acid receptors and the 2/5-fold wall of CPV and FPV serves as the transferrin receptor binding site [41,46]. The 2/5-fold region also contains determinants of tissue tropism for MVM and pathogenicity for PPV [20]. As previously mentioned, the 2/5-fold wall along with the 3-fold protrusions serve as dominant antigenic epitopes for the parvoviruses. The differences observed for BuV1 at these regions suggests a potential similar role in antigenic reactivity for the BuVs. Whether or not the 2-fold region will serve as a glycan receptor attachment site remains to the determined. Finally, the 5-fold axis and its surrounding region, are structurally conserved for the protoparvoviruses, although the apex of the HI loop of BuV1 VP2 is conformationally different (Figure 6A). This causes a difference on the floor around the channel, but the channel formed by the DE loop and thus the channel itself is conserved (Figure 6B). This is likely due to its proposed function in genome packaging and uncoating as well as PLA2 externalization for endo/lysosomal pathway escape during cell infection by the parvoviruses [47].

### 3.4. BuV1 Comparison to Other Human Parvoviruses Suggests Host Specific Capsid Structural Evolution

**Figure 7:**
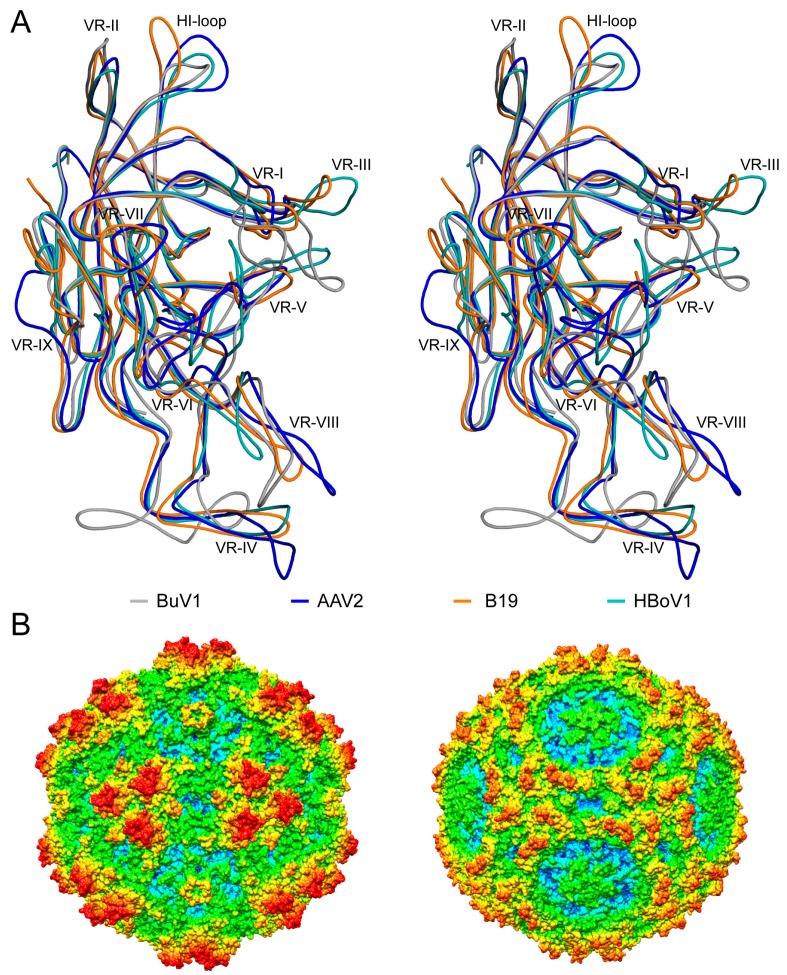
The structures of human infecting parvoviruses. (**A**) Stereo projection of the structural superposition of BuV1 VP2 onto previously described human infecting parvovirus structures shown in coil representation. Colors are as indicated. The VR regions, VR-I to VR-IX, and the HI-loop are labeled. This image was generated using the program PyMOL (DeLano, W. L. (2002). PyMOL. DeLano Scientific, San Carlos, CA, USA, 700); (**B**) radially-colored capsid surface representations (blue to red, as shown in Figure 1) of the BuV1 (left) and Parvovirus B19 (right) capsid models viewed along the 2-fold axis. This image was generated using Chimera [33].

**Figure 8:**
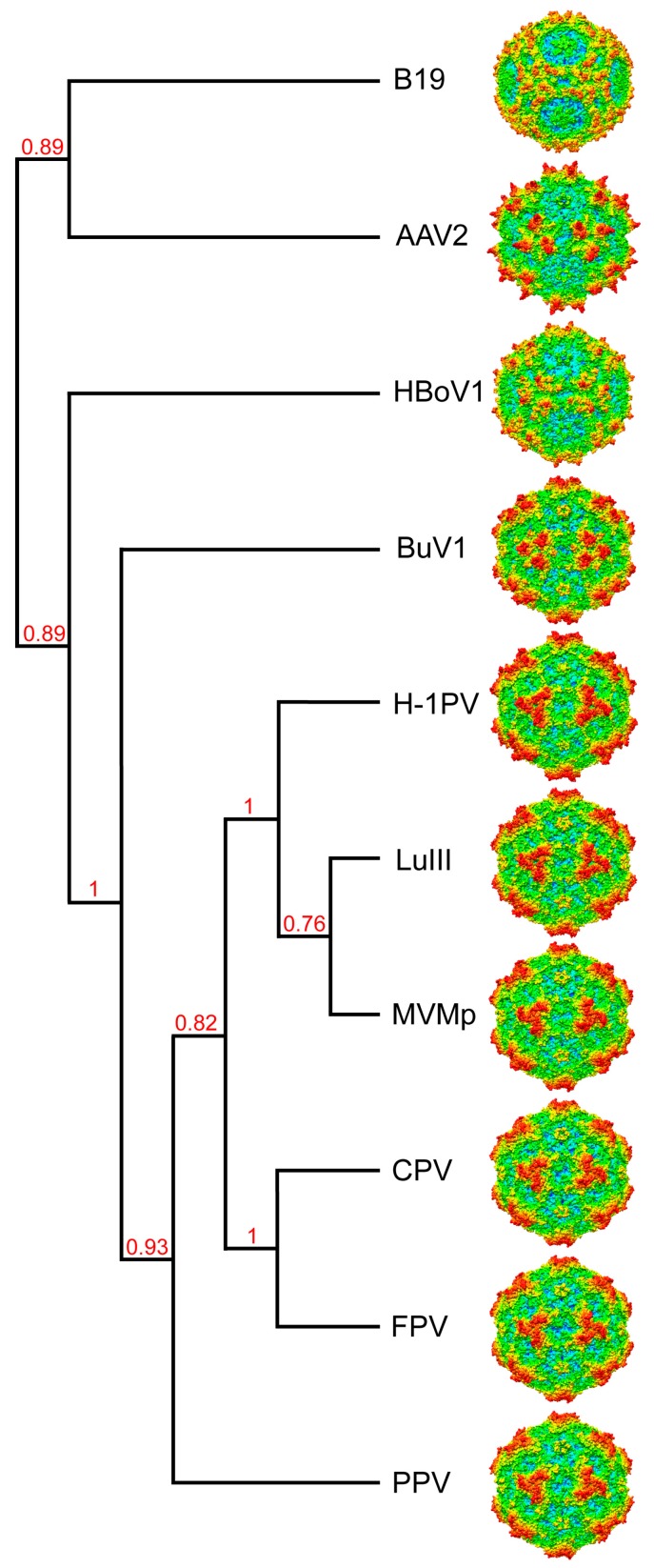
Cladogram showing the relationship between selected members of the *Parvovirinae* subfamily. This image was generated online (http://www.phylogeny.fr/) utilizing the VP2/3 sequences as input. Branch support values are given. Radially-colored capsid surface representations (blue to red, as shown in Figure 1) are viewed along the 2-fold axis and generated using Chimera [33].

A comparison of BuV1 to other human infecting parvoviruses, for example, Adeno-associated virus serotype 2 (AAV2), Parvovirus B19, and HBoV1, revealed very low sequence identity, ~15–24%, among all the four viruses (Table 3). However, as observed for the protoparvovirus comparison, the structural similarity was much higher at 44 to 63% (Table 3). The overall VP2 structure topologies of these viruses were similar, with the core being completely superposable (Figure 7A). Divergence is observed once again in the previously defined common VRs. As an example, the loops at the 3-fold axes are conserved among the human viruses. However, loops extending from the conserved regions adopted drastically different conformations to form the 3-fold protrusions (Figure 7A). The largest differences occurred at VR-I and VR-VIII, with ~20 residues inserted in BuV1 compared to AAV2, with smaller insertions compared to the other viruses, and at VR-IV, where a conformation switch positions the majority of this loop at the 2/5-fold wall of BuV1 instead of the top of the protrusion (Figure 7A). Interestingly, VR-V was unique in AAV2 (Figure 7A). These and other VR differences, arising from residue deletions/insertions and conformational changes, alter the local surface morphologies of the capsids while maintaining the general parvovirus features at the 2-, 3-, and 5-fold axes (Figure 7B and Figure 8). Importantly, despite these differences, all the human infecting viruses had a separated 3-fold protrusion arrangement, not a single pinwheel. This observation suggests host specific interaction(s) in humans involving regions of the 3-fold protrusions.

Significantly, both AAV2 and B19 utilize amino acids located on the loops forming the 3-fold protrusions to interact with cell surface glycan receptors [48,49,50]. The 3-fold region is also responsible for interaction with antibodies in AAV2 and HBoV1, while the 2/5-fold wall interacts with antibodies for AAV2 and B19 [42,44,49,51]. For AAV2 and other dependoparvoviruses, the 2/5-fold wall and 3-fold protrusions serve as sites of common antigenic diversity [42,43,52]. For HBoVs, the 3-fold protrusions determine strain specific antibody recognition, while antibodies directed against the 5-fold is cross-reactive among HBoV strains [44]. A similar functional utilization of these capsid regions is yet to be determine for the BuVs.

## 4. BuVs Structures Conserve Functional Parvovirus Features

The comparative analysis of BuV1 with members of the protoparvoviruses and human infecting parvoviruses from three different genera supports previous observations that parvoviruses have evolved to conserve their VP structure, regardless of sequence. This suggests a need to maintain a specific VP topology to assemble a specific capsid morphology to enable functions required for infectivity. The majority of the residues that are identical among the viruses compared form their core β-barrel and αA regions. This suggests that instructions to fold a similar VP topology is contained within the diverse sequence of each member of the *Parvoviridae* genera. The commonality between these viruses is single-stranded (ss) DNA binding and packaging which may play a role in the folding of this VP topology.

A cladogram generated for BuV1 as well as the protoparvoviruses and human infecting parvoviruses compared based on the VP2 sequences places BuV1 between the animal and human viruses (Figure 8). Consistent with this observation, despite the higher (~10%) BuV1 VP2 sequence identity to the protoparvoviruses, the three separated protrusion 3-fold morphology of the BuVs resembled the human infecting viruses rather than the single pinwheel of the protoparvoviruses (Figure 8). The fact that the 3-fold region serves as glycan receptor binding sites for two of the viruses compared, AAV2 and B19, suggests a similar function for the BuVs. However, the 2-fold depression also serves as the glycan receptor binding sites for protoparvoviruses. Thus, whether these regions serve similar receptor attachment sites for the BuVs remains to be determined.

The BuVs are reported to be antigenically distinct. The capsid region that differs the most between them is the 2/5-fold wall containing VR-IV, VR-VII and VR-IX. These regions are antigenically dominant for the AAVs as well as for CPV. A role for this and other regions in antibody recognition remains to be determined for the BuVs. The structures reported here will facilitate this analysis.

## 5. Conclusions

The study reports the first structures determined for human-infecting members of the *Protoparvovirus* genus, which until recently, contained only animal infecting members. Significantly, while the structure conserved the VP topology of other members of this genus, a deletion and conformational difference at one of the loops forming the 3-fold region results in capsid surface morphology that resembles the other human-infecting parvoviruses from other genera. This suggests a host-specific function for the separated arrangement of the protrusions surrounding the 3-fold region. These structures will aid future efforts at functional annotation.

## Figures and Tables

**Table 1:** Summary of data collection, image-processing parameters, and refinement statistics.

Processing and Refinement Parameters	BuV1	BuV2	BuV3
Total number of micrographs	1961	429	689
Defocus range (µm)	0.79–4.42	0.90–3.87	1.82–3.34
Electron dose (e^−^/Å^2^)	59.5	57.2	75
Frames/micrograph	60	34	50
Pixel size (Å/pixel)	1.07	1.22	1.06
Startin gnumber of particles	59,170	8404	6543
Particles used for final map	29,596	7564	5234
B-factor used for final map (Å^2^)	−50	−100	−100
Resolution of final map (Å)	2.84	3.79	3.25
PHENIX Model Refinement Statistics			
Residue range	33–568	33–567	33–572
Map correlation coefficient	0.863	0.678	0.852
RMSD (root-mean-square deviation) [bonds] (Å)	0.01	0.01	0.01
RMSD [angles] (Å)	0.91	1.20	0.97
All-atom clash score	10.41	18.44	11.79
Ramachandran Plot			
Favored (%)	95.5	94.0	94.8
Allowed (%)	4.5	6.0	5.2
Outliers (%)	0	0	0
Rotameroutliers (%)	0	0	0
C-β deviations	0	0	0

**Table 2:** Sequence identity and structural similarity among the protoparvoviruses.

	BuV1	MVMp	LuIII	H-1PV	CPV	FPV	PPV	
BuV1		75.0	76.7	74.1	74.3	74.8	75.7	Structural similarity(%)
MVMp	29.5		97.8	97.8	90.7	90.5	91.4	
LuIII	31.4	73.2		97.1	89.3	88.0	91.7	
H-1PV	29.9	67.3	67.7		90.9	90.4	92.0	
CPV	30.8	51.9	50.7	51.2		96.7	91.1	
FPV	31.0	51.9	50.7	51.2	99.7		90.0	
PPV	31.3	49.4	50.3	49.3	57.2	57.2		
	VP2/3 sequence identity (%)							

**Table 3:** Sequence identity and structural similarity among the human infecting parvoviruses.

	BuV1	AAV2	B19	HBoV1	
BuV1		54.7	44.8	48.3	Structural similarity (%)
AAV2	18.2		62.8	63.2	
B19	15.5	23.7		51.6	
HBoV1	19.0	24.0	20.6		
	VP2/3 sequence identity (%)				
